# Leukemia-associated gene *MLAA-34* reduces arsenic trioxide-induced apoptosis in HeLa cells via activation of the Wnt/β-catenin signaling pathway

**DOI:** 10.1371/journal.pone.0186868

**Published:** 2017-10-23

**Authors:** Pengyu Zhang, Xuan Zhao, Wenjuan Zhang, Aili He, Bo Lei, Wanggang Zhang, Yinxia Chen

**Affiliations:** 1 Department of Hematology, Second Affiliated Hospital, Xi' an Jiaotong University, Xi'an, Shaanxi, China; 2 Department of Histology&Embryology, Xi'an Medical College, Xi'an, Shaanxi, China; 3 Shaanxi Blood Center, Xi'an, Shaanxi, China; National Cancer Center, JAPAN

## Abstract

Our laboratory previously used the SEREX method in U937 cells and identified a novel leukemia-associated gene *MLAA-34*, a novel splice variant of *CAB39L* associated with acute monocytic leukemia, that exhibited anti-apoptotic activities in U937 cells. Whether *MLAA-34* has an anti-apoptotic role in other tumor cells has not yet been reported. We explored whether *MLAA-34* exhibited anti-apoptotic effects in HeLa cervical cancer cells and the possible mechanism of action. We generated a HeLa cell line stably expressing *MLAA-34* and found that *MLAA-34* overexpression had no effect on the growth, apoptosis and cell cycle of HeLa cells. However, upon treatment with arsenic trioxide (ATO) to induce apoptosis, the cell viability and colony formation ability of ATO-treated *MLAA-34* stable HeLa cells were significantly higher than that of ATO-treated controls, and the apoptosis rate and proportion of G_2_/M cells also decreased. We found that ATO treatment of HeLa cells resulted in significant decreases in the expression of *β-catenin* mRNA and protein and the downstream target factors c-Myc, cyclin B1, and cyclin D1 in the Wnt signaling pathway. Notably, ATO-treated *MLAA-34* stable HeLa cells showed a significant reduction in the ATO-mediated downregulation of these factors. In addition, *MLAA-34* overexpression significantly increased the expression of nuclear β-catenin protein in ATO-treated cells compared with HeLa cells treated only with ATO. Thus, here we have found that the Wnt/β-catenin signaling pathway is involved in ATO-induced apoptosis in HeLa cells. *MLAA-34* reduces ATO-induced apoptosis and G_2_/M arrest, and the anti-apoptotic effect may be achieved by activating the Wnt/β-catenin signaling pathway in HeLa cells.

## 1. Introduction

We previously used serologic analysis of a recombinant cDNA expression library (SEREX) in U937 acute monocytic leukemia cells to identify leukemia-associated antigens. Using this approach, we identified a novel leukemia-associated gene, *MLAA-34* [1], which is a novel splice variant of *CAB39L* that is associated with acute monocytic leukemia [2]. At present, little is known about the function of the *CAB39L* gene. A previous study identified *MLAA-34* as a novel anti-apoptotic gene in U937 cells [2]. Clinical research has shown that *MLAA-34* is highly expressed in primary and recurrent acute mononuclear cell leukemia (M5) patients, while it shows low expression in M5 patients with complete remission [3]. Examination of *MLAA-34* in 30 kinds of tumor cell lines, 11 kinds of non-tumor cell lines and 5 kinds of hybrid cells revealed high expression of *MLAA-34* in U937 and MHCC97-H cells and peripheral blood mononuclear cells in patients with M5 [4]. The expression was relatively weak in other leukemia and lymphoma cell lines, as well as in some solid tumor cell lines; almost no expression was detected in non-tumor cell lines. *MLAA-34* gene was not expressed in the HeLa cervical cancer cell line. Further study found that the anti-apoptotic effect of *MLAA-34* in U937 cells may be related to activation of the Wnt/β-catenin signaling pathway [5]. So far, research on the function of *MLAA-34* gene has been limited to the acute monocytic leukemia cell line U937. However, whether *MLAA-34* has an anti-apoptotic role in other tumor cells has not yet been reported.

Arsenic trioxide (ATO) has been successfully used to treat acute promyelocytic leukemia and has also been researched as a possible treatment for other hematological and solid cancers. ATO has shown therapeutic efficacy in the treatment of cervical cancer and has been demonstrated to effectively induce apoptosis of cervical cancer cells at low concentrations *in vitro* [6, 7].

In this study, we explored the effect of *MLAA-34* expression on apoptosis and the cell cycle of HeLa cells treated with ATO and investigated whether the mechanism of action is related to the Wnt/β-catenin signaling pathway.

## 2. Materials and methods

### 2.1. Cell culture

HeLa cells were obtained from the Institute for Cancer Research, the School of Life Science and Technology, Xi’an Jiaotong University (China). Cells were cultured in DMEM medium with 10% fetal calf serum. In all experiments, cells were used in the log-growth phase. ATO was diluted in medium and the concentration of ATO in experiments was 1 μmol/L.

### 2.2. Construction of the *MLAA-34* recombinant lentiviral vector and virus production

The recombinant vector containing *MLAA-34* cDNA was constructed using the lentiviral expression vector pGC-LV (GeneChem Limited Company. Shanghai, China); this vector was named pGC-FU-MLAA-34. The vector that does not contain the *MLAA-34* cDNA was termed as pGC-FU. The construction of the recombinant lentiviral vector, virus packaging and virus titer determination method were performed as previously described [4].

### 2.3. Establishment of *MLAA-34* overexpressing stable cell lines

HeLa cells were cultured in a 6-well tray in DMEM supplemented with 10% FBS. The recombinant *MLAA-34* lentivirus was added to HeLa cells at multiplicity of infections (MOIs) of 30, 50 and 100 with ENi.S and 5 μg/ml polybrene when the fusion degree is about 70%. The expression of green fluorescence was observed by fluorescence microscope after 72 h, and the optimal infection rate was determined. The infection rate was determined by flow cytometry.

Recombinant *MLAA-34* lentivirus was used to infect HeLa cells at the optimal MOI, and the culture medium were replaced with fresh medium after 24 h. Stably infected clones were established by selection with G418 (Sigma-Aldrich, St. Louis, MO) at a concentration of 600 μg/ml for 3 weeks. Resistant clones were classified and expanded by culture; the expression of GFP was confirmed by fluorescence microscopy to determine the efficiency of infection. The expression level of *MLAA-34* gene and protein was detected by RT-PCR and western blot assay. After 5 weeks of extended culture post-G418 selection, we obtained stable highly expressing *MLAA-34* HeLa cell lines.

### 2.4. RNA analysis

Total RNA was isolated from cells using an acid guanidinium-phenol-chloroform method (Trizol, Invitrogen). RT-PCR was carried out using the HighFidelity PrimeScriptTM RT-PCR Kit (Takara, DaLian, China). Primers for analysis were designed using Primer Premier Software (PREMIER Biosoft International, Palo Alto, USA) based on known sequences. Primer sequences used for amplification are listed in Table 1. Cycling parameters for the reactions were as follows: 95°C for 3 min, 30 cycles of 98°C for 10s, 55°C for 30 s, 72°C for 1 min, and a final extension at 72°C for 10 min. PCR products were analyzed by 2% agarose gel electrophoresis. Quantity One 4.6.2 software was used to analyze the gray value of electrophoresis results.

**Table 1:** PCR primers.

Gene	Primer	Amplification length (bp)
*MLAA-34*	5'-TCTGTGTGGAACGAACGACAA—3'(Up)	105
	5'-TGACACTCATAGCTGACCTGCA-3'(Down)	
*β-catenin*	5'-GGAAGGGATGGAAGGTCTC-3'(Up) 5'-GGGATGGTGGGTGTAAGA G-3'(Down)	323
*β-actin*	5'-CCTGTACGCCAACACAGTGC-3'(Up)	211
	5'-ATACTCCTGCTTGCTGATCC- 3'(Down)	

### 2.5. Western blot analysis

Cells were collected in 80 μl lysis buffer containing protease inhibitors (Beyotime, China). Nuclear proteins were extracted using the CelLytic™ NuCLEAR™ Extraction Kit (Sigma, USA). Protein concentration was determined using the BCA kit (Sigma, USA). *MLAA-34* is a novel splice variant of the *CAB39L* gene, but with the same coding frame, encoding the same protein, therefore the CAB39L monoclonal antibody (Santa Cruz Biotechnology, sc-100390), which can recognize MLAA-34 protein, served as MLAA-34 antibody. Other antibodies were used as follows: anti-β-actin (Sigma-Aldrich), anti-β-catenin (Santa Cruz Biotechnology), anti-c-Myc (Abcam, UK), anti-cyclin B1 (Santa Cruz Biotechnology), anti-cyclin D1 (Santa Cruz Biotechnology), anti-Histone H3 (Sigma-Aldrich), HRP-labeled Goat Anti-Mouse IgG (Beyotime, China), and HRP-labeled Goat Anti-Rabbit IgG (Beyotime, China).

### 2.6. Cell viability assay

Cells (3×10^3^) were plated on a 96-well plate and allowed to adhere overnight. The treatment group was treated with ATO to a final concentration of 1 μmol/L, and then cultured for 1, 2, 3, 5 and 7 d. MTT assays were then carried out. Absorbance was measured at 540 nm. All assays were performed in triplicate and were repeated three times under independent conditions. Data are presented as means±SD. Cell viability was calculated as % of control cells, and the viability of HeLa cells (control group) was considered 100%.

### 2.7. Flat plate clone formation assay

Cells in logarithmic growth phase were digested into a single cell suspension with trypsin-EDTA solution and then 5 ml of the suspension was seeded into 25 ml cell culture flasks at a density of 40 cells/ml. The treatment group was treated with ATO at a final concentration of 1 μmol/L, and the culture medium was replaced with fresh medium after 24 h. Cells were cultured for about 3 weeks and colony formation was monitored under a magnification of 100 x. The assay was done in triplicate.

### 2.8. Analysis of cell cycle and apoptosis

Cells were seeded into a six-well tissue culture plate. The treatment group was treated with ATO at final concentration of 1 μmol/L. After 48 h, cells were harvested. Cell cycle analysis was performed as previously described [2]. Annexin V (Ann-V) and propidium iodide (PI) staining were performed using the Annexin V-FITC Apoptosis Detection Kit (BD Biosciences), followed by flow cytometric analysis. All assays were performed in triplicate and were repeated three times under independent conditions. Relative G_2_/M cell growth rate = (G_2_/M cells in ATO treatment group—G_2_/M cells in control group)/G_2_/M cells in control group×100%.

### 2.9. Cignal TCF/LEF reporter assay

The Cignal TCF/LEF reporter assay (Qiagen, Dusseldorf, Germany) was performed according to the manufacturer’s instructions. After preparation of complex formation according to the instructions, suspended cells were obtained from exponential phase of growth cells (4×10^5^/ml, 100 μl/well). The 96-well plates were cultured at 37°C with 5% CO_2_ and16 hours later, the medium was replaced with assay medium (Opti-MEM® containing 0.5% fetal bovine serum, 1% NEAA, 100 U/ml penicillin and 100 μg/ml streptomycin).The next day, ATO was added and the cells were cultured for another 48 h. Luciferase assays were then carried out using the Dual-Luciferase Reporter Assay System (Promega, WI, USA). The TCF/LEF reporter activity is presented as the relative ratio of Firefly luciferase activity to Renilla luciferase activity. All experiments were performed three times with triplicate replicates.

### 2.10. Statistical analysis

Statistical significance was assessed by comparing mean (±SD) values with Student’s t-test for independent groups. *P* < 0.05 was considered statistically significant. All analyses were performed using SPSS software.

## 3. Results

### 3.1. Establishment of stable HeLa cell lines with *MLAA-34* overexpression

**Fig 1:**
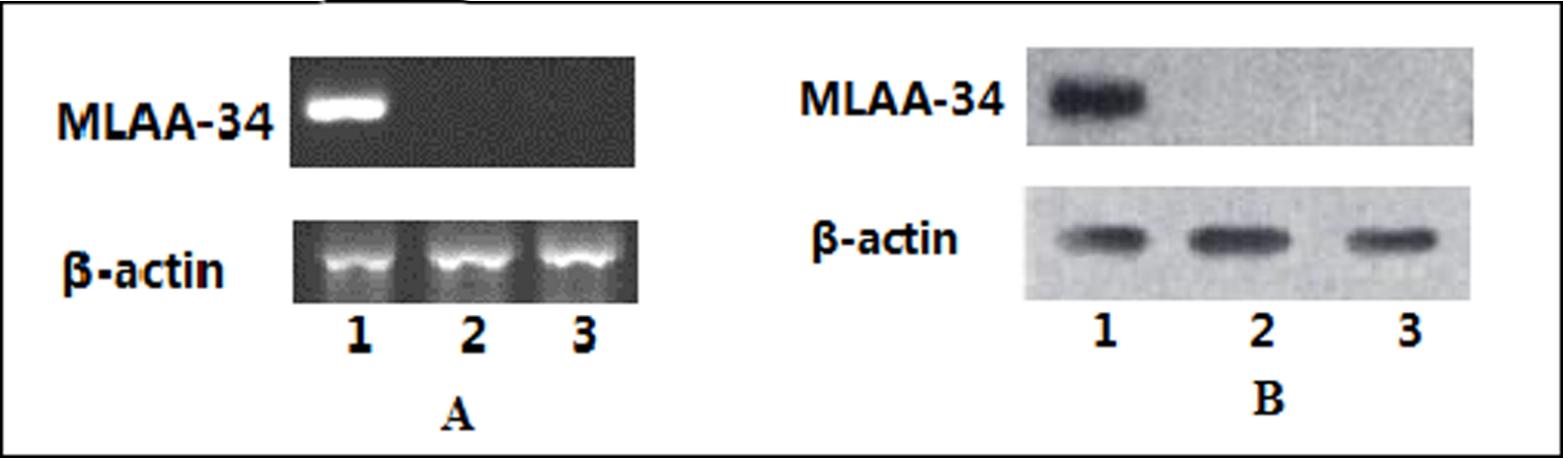
Expression of the *MLAA-34* gene in HeLa cells. *MLAA-34* mRNA (A) and protein (B) levels were determined in HeLa cell groups using RT-PCR and western blot. Lane 1. pGC-FU-MLAA-34 vector transfection group, Lane 2. PGC-FU vector transfection group, Lane 3. HeLa cells.

To examine the function of *MLAA-34* in HeLa cells, we generated HeLa cell lines stably overexpressing *MLAA-34* using our previously published lentivirus vector (pGC-FU-MLAA-34) [4]. The positive clones were selected by G418 for 3 weeks, and then after 5 weeks of extended culture, we confirmed highly expressing *MLAA-34* HeLa cell lines. As shown in Fig 1 (S1–S4 Figs), the *MLAA-34* stable HeLa cell lines showed increased *MLAA-34* mRNA (Fig 1A) and protein (Fig 1B) levels as compared to control and empty vector-transfected cells, confirming that the exogenous *MLAA-34* gene was stably expressed in HeLa cells.

### 3.2. *MLAA-34* overexpressing stable HeLa cells showed increase cell viability under ATO treatment

We next examined the cell viability in response to ATO treatment using MTT assays. In the absence of ATO treatment, the cell viability of the *MLAA-34* stable cell lines compared with the control cell lines was not statistically different (Table 2), indicating that overexpression of *MLAA-34* had no impact on HeLa cell viability. Upon treatment with ATO (1 μmol/L), the cell viability markedly decreased in all groups over time. Notably, the cell viability of *MLAA-34* overexpressing stable cells treated with ATO was significantly higher than that of the control cells treated with ATO. This showed that overexpression of *MLAA-34* could block, in part, the inhibitory effects of ATO on cell viability in HeLa cells.

**Table 2:** Effects of ATO on the viability of HeLa cells.

Group	Viability(%)				
	1d	2d	3d	5d	7d
HeLa	100	100	100	100	100
pGC-FU	98.6±3.2	102.3±2.7	97.6±3.6	95.8±2.8	96.3±2.1
pGC-FU-MLAA-34	99.6±4.5	98.6±1.2	101.5±1.7	98.4±2.6	97.4±2.5^☆^
ATO+ HeLa	82.3±2.8	72.3±0.9	63.9±3.5	60.2±2.6	58.2±1.6
ATO+pGC-FU	83.5±3.2	71.7±1.6	64.9±2.8	59.5±3.2	57.6±2.3^△^
ATO+ pGC-FU-MLAA-34	96.4±2.5	93.7±1.8	84.5±3.5	80.6±1.8	78.6±2.3*

Data are presented as means±SD of three independent experiments.

^☆^*P* > 0.05 compared with pGC-FU cells

^△^*P* > 0.05 compared with ATO+HeLa cells

*P < 0.05 compared with ATO+pGC-FU cells.

### 3.3. *MLAA-34* overexpressing stable HeLa cells showed increased colony formation ability under ATO treatment

**Fig 2:**
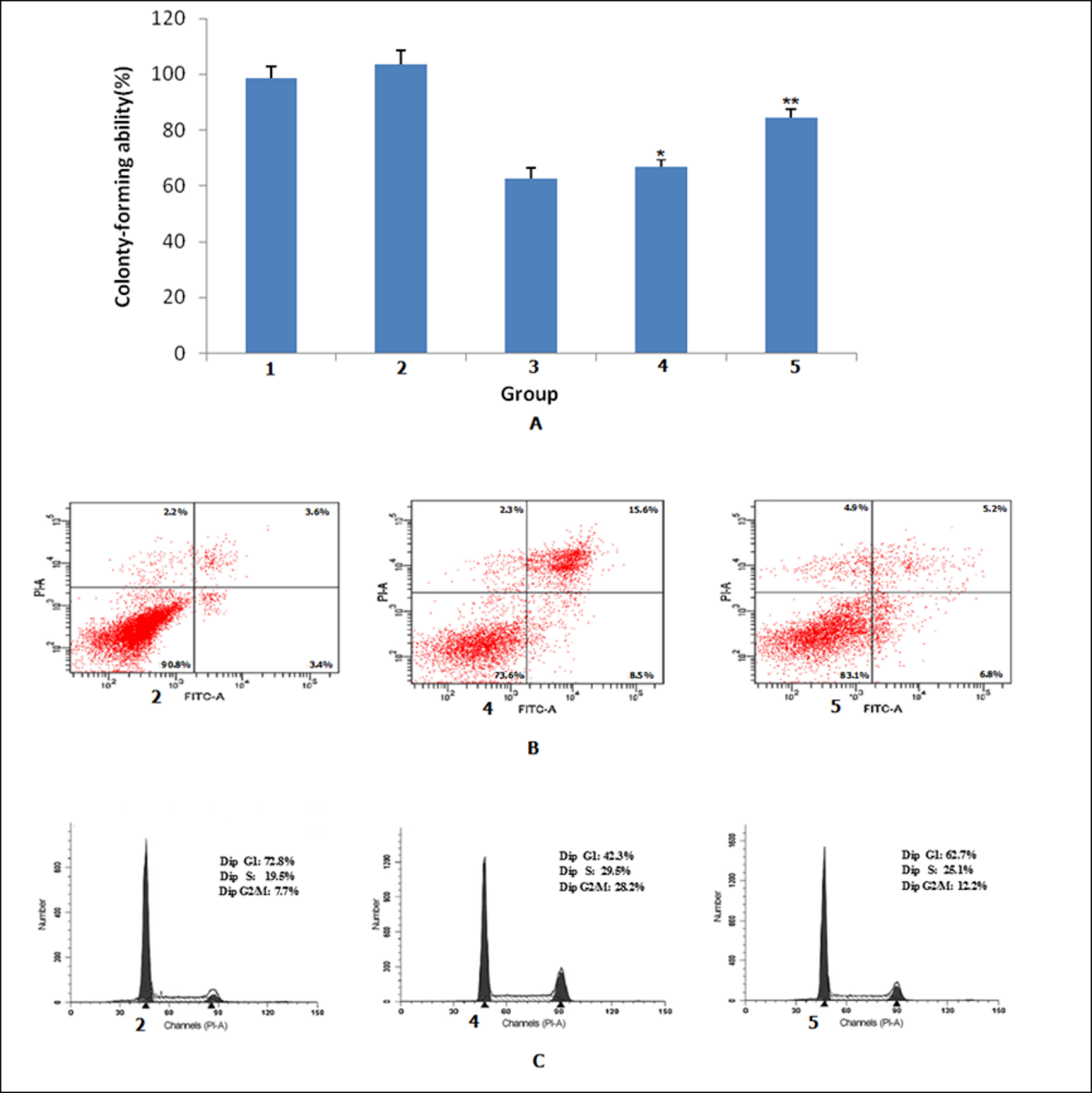
Effects of MLAA-34 on the colony formation ability, apoptotic rate and cell cycle distribution of HeLa cells. **(**A) Colony-forming rate was determined relative to the rate of untreated HeLa cells, which was normalized to 100%. Data are presented as means±SD of three independent experiments. Cells were treated with ATO (1 μmol/L) for 48 h and then harvested for flow cytometric analysis to determine (B) cell apoptotic rate and (C) cell cycle distribution. Data are representative of three independent experiments. *P>0.05, compared with ATO+HeLa; **P < 0.01, compared with ATO+HeLa. 1: pGC-FU, 2: pGC-FU-MLAA-34, 3: ATO+HeLa, 4: ATO+pGC-FU, 5: ATO+ pGC-FU-MLAA-34.

We next examined colony formation ability of the HeLa cell lines with ATO treatment. In the untreated cell groups, the colony formation ability of control cells and *MLAA-34* stable cells was 98.5%±4.2% and 103.4%±5.3% respectively, with no statistical significance between the two groups (Fig 2A). Upon treatment with ATO (1 μmol/L), the colony formation ability of all cell lines markedly decreased. The colony formation abilities of both ATO-treated control groups were 62.5%±3.9% and 66.7%±2.8%. However, the colony formation ability of *MLAA-34* stable cells treated with ATO was 84.3%±3.3%, and this was significantly higher compared with the ATO treated control group. These findings showed that *MLAA-34* overexpression blocked, in part, the inhibition of colony formation ability by ATO treatment.

### 3.4. Effects of *MLAA-34* overexpression on cell cycle distribution and apoptosis of HeLa cells under ATO treatment

We next examined the apoptosis rate and cell cycle distribution of the HeLa cell lines (Table 3). The *MLAA-34* stable cells showed no significant difference in apoptosis or cell cycle distribution compared to control HeLa cells, suggesting that overexpression of the *MLAA-34* gene had no significant effects on apoptosis and cell proliferation. Upon treatment with ATO, the apoptosis rate of the control group was 21.8%, while the apoptosis rate of the *MLAA-34* overexpression stable cell line was 10.8% (Fig 2B), indicating that *MLAA-34* overexpression significantly reduced the apoptosis induced by ATO. In line with this observation, the relative G_2_/M cell growth rate of the control group treated with ATO was 194.9%, while that of the *MLAA-34* overexpression stable cell line treated with ATO was only 56.5%, indicating that *MLAA-34* overexpression significantly reduced the increased numbers of cells in G_2_/M phase induced by ATO (Fig 2C). These results show that upon induction of apoptosis by ATO in HeLa cells, *MLAA-34* overexpression had an anti-apoptotic effect and reduced the G_2_/M phase arrest.

**Table 3:** Effects of ATO on the cell cycle distribution and apoptotic in the HeLa cell.

Group	Apoptotic (%)	G1	S	G_2_/M	Relative G_2_/M cell growth rate (%)
HeLa	6.4±1.8	72.5±3.4	17.6±4.5	9.9±2.0	-
pGC-FU	6.7±2.0	73.4±2.8	16.5±3.5	10.1±3.5	-
pGC-FU-MLAA-34	5.4±3.2	73.6±1.6	17.8±2.8	8.6±3.2	-
ATO+HeLa	22.6±2.8	41.3±3.0	28.9±1.7	29.8±2.3	201.1
ATO+pGC-FU	21.8±3.2	40.2±2.5	30.6±3.3	29.2±1.7*	194.9
ATO+pGC-FU-MLAA-34	10.8±1.9	63.2±1.7	21.3±4.0	15.5±3.2**	56.5

Data are presented as means±SD of three independent experiments.

*P < 0.01 compared with pGC-FU cells

**P < 0.05 compared with ATO+pGC-FU cells.

### 3.5. Effects of ATO on the Wnt/β-catenin signaling pathway in U937 cells

**Fig 3:**
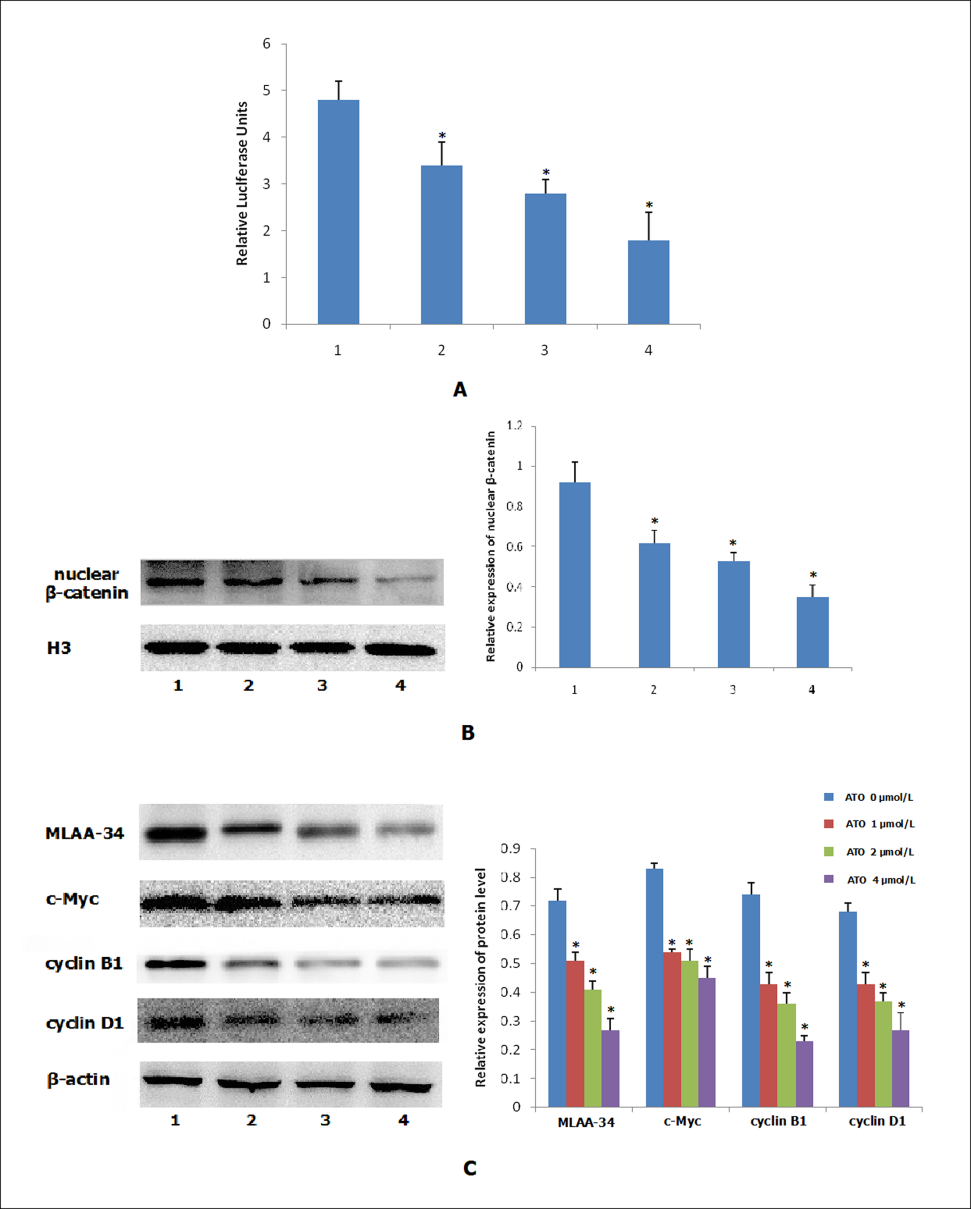
ATO inhibits the activity of the Wnt/β-catenin pathway in U937 cells. Cells were treated with ATO (0, 1, 2, 4 μmol/L) for 48 h, and then harvested for analyses. (A) The Cignal LCF/TCF reporter assay. (B) Western blot analysis for β-catenin expression in nuclear extracts of cells. (C) Western blots analysis of MLAA-34, c-Myc, cyclin B1 and cyclin D1 protein. Data are presented as means±SD of three independent experiments. *P < 0.05, compared with control U937 cells (ATO 0μmol/L). 1: ATO 0 μmol/L, 2: ATO 1 μmol/L, 3: ATO 2 μmol/L, 4: ATO 4 μmol/L.

Because the Wnt/β-catenin signaling contributes to the development of U937 cells, and U937 cells could be induced apoptosis by ATO, we investigated the Wnt/β-catenin signaling pathway in U937 cells following exposure to ATO. U937 Cell lines were treated with ATO (0, 1, 2, 4 μmol/L) and then cultured for 48 h, the Cignal LCF/TCF reporter assay was used to evaluate the activity of Wnt/β-catenin signaling. As shown in Fig 3A, Different concentrations of ATO could significantly inhibit the transcriptional activity of the TCF/LEF reporter vector compared with untreated U937 cells (*P*<0.05), and in a dose-dependent manner. Moreover, western blot results showed that the expression of nuclear β-catenin in ATO-treated cells was significantly decreased compared with control U937 cells (Fig 3B, S5 and S6 Figs). We further assessed the expression of MLAA-34 protein, as well as the expression of proteins of Wnt signaling pathway downstream gene c-Myc, cyclin B1 and cyclin D1. As shown in Fig 3C (S7–S11 Figs), ATO decreased the protein expression of MLAA-34, c-Myc, cyclin B1 and cyclin D1 in a dose-dependent manner (P<0.05). These results indicate that ATO reduces the expression of MLAA-34 and inhibits the activity of the Wnt/β-catenin signaling pathway in U937 cells.

### 3.6. Effects of *MLAA-34* overexpression on the Wnt/β-catenin signaling pathway in Hela cells

**Fig 4:**
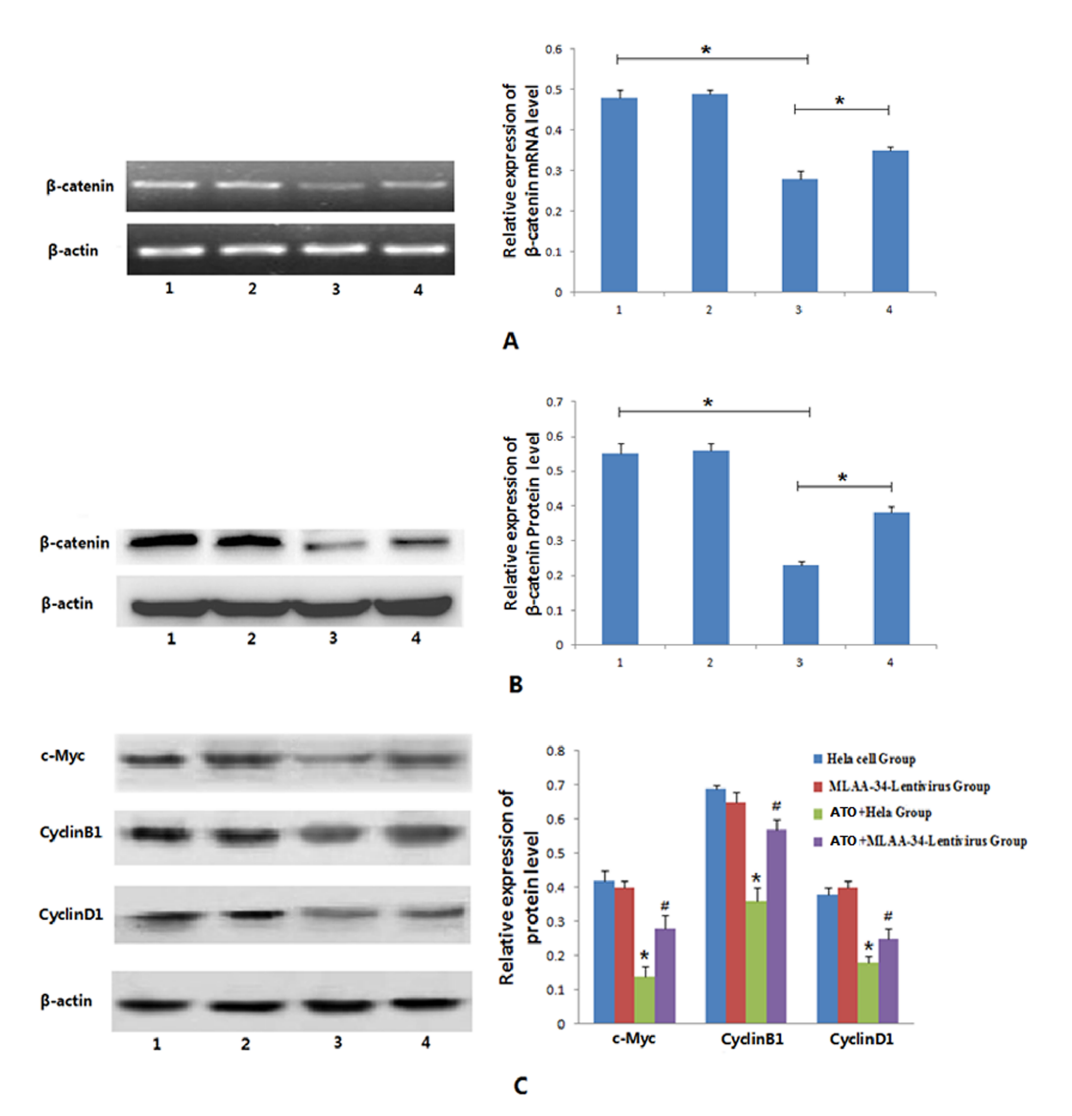
Effects of MLAA-34 on the levels of β-catenin, c-Myc, cyclin B1 and cyclin D1 in HeLa cells. Cells were treated with ATO (1 μmol/L) for 48 h, and then harvested for analyses. (A) RT-PCR analysis of *β-catenin* mRNA levels in all cell groups. (B) Western blot analysis of β-catenin protein. (C) Western blots analysis of c-Myc, cyclin B1 and cyclin D1 protein. Data are presented as means±SD of three independent experiments. A and B: *P < 0.05; C: *P < 0.05, compared with HeLa cells; #*P* < 0.05, compared with ATO+HeLa cells. 1: HeLa cells, 2: pGC-FU-MLAA-34 cells, 3: ATO+HeLa cells, 4: ATO+ pGC-FU-MLAA-34 cells.

We next investigated whether the anti-apoptotic effect of the *MLAA-34* gene was related to the Wnt/β-catenin signaling pathway in HeLa cells. Cell lines were treated with ATO (1 μmol/L) and then cultured for 48 h. We assessed the expression of *β-catenin* (the Wnt signaling pathway key gene) mRNA and protein, as well as the expression of proteins of Wnt signaling pathway downstream genes c-Myc, cyclin B1 and cyclin D1. As shown in Fig 4 (S12–S19 Figs), in the absence of ATO treatment, *MLAA-34* overexpression had no impact on the mRNA and protein expression of *β-catenin* or the protein expression of c-Myc, cyclin B1 and cyclin D1. These results were consistent with the fact that *MLAA-34* overexpression did not affect the growth curve, apoptosis rate and cell cycle of HeLa cells without ATO treatment. Upon treatment with ATO, the mRNA and protein expression of *β-catenin* and c-Myc, cyclin B1 and cyclin D1 protein expressions were significantly decreased compared with untreated cells, and the difference was statistically significant (*P* < 0.05). This result suggests that the induction of apoptosis by ATO in HeLa cells may be partly achieved by inhibiting the Wnt/β-catenin signaling pathway. Furthermore, in the *MLAA-34* stable cells treated with ATO, the mRNA and protein expression of *β-catenin* and c-Myc, cyclin B1 and cyclin D1 protein expressions were significantly higher than those of the ATO-treated control group, and the difference was statistically significant (*P* < 0.05); however, these levels were still lower than those of untreated HeLa cells. These results suggest the inhibitory effect of MLAA-34 on ATO induced HeLa cell apoptosis may be partially achieved through its blocking of ATO-mediated disruption of the Wnt/β-catenin signaling pathway.

**Fig 5:**
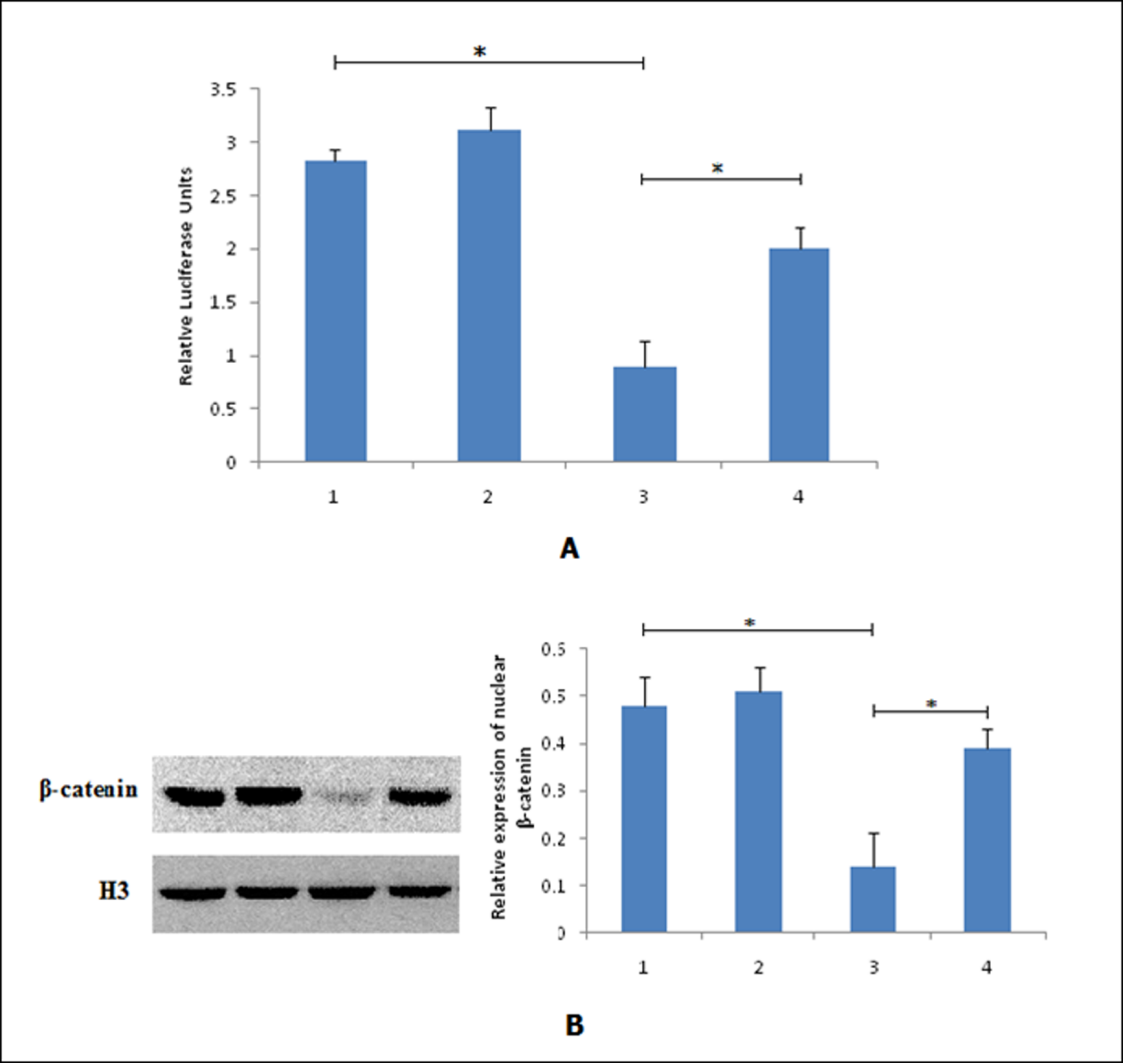
MLAA-34 reduces ATO-mediated inhibition of the Wnt/β-catenin pathway in HeLa cells. (A) *MLAA-34* stable HeLa cells were transfected with the Cignal TCF/LEF reporter plasmid and treated with ATO for 48 h; the reporter activities were determined by a luciferase assay. (B): Western blot analysis for β-catenin expression in nuclear extracts of HeLa cells. Cells were treated with ATO (1 μmol/L) for 48 h before analyses. Data are presented as means±SD of three independent experiments. A and B: *P < 0.05. 1: HeLa cells, 2: pGC-FU-MLAA-34 cells, 3: ATO+HeLa cells, 4: ATO+ pGC-FU-MLAA-34 cells.

To validate the involvement of the Wnt/β-catenin signaling cascade, the Cignal LCF/TCF reporter assay was used to evaluate the activity of Wnt/β-catenin signaling. As shown in Fig 5A, ATO (1 μmol/L) treatment could significantly inhibit the transcriptional activity of the TCF/LEF reporter vector compared with untreated HeLa cells (0.89±0.25, *P* < 0.05). ATO treatment in *MLAA-34* overexpressing stable cells significantly increased the TCF/LEF reporter activity compared with control HeLa cells treated with ATO (2.01±0.19, *P* < 0.05). Furthermore, western blot results showed that the expression of nuclear β-catenin in ATO-treated cells was significantly decreased compared with untreated HeLa cells. In contrast, the expression of nuclear β-catenin in *MLAA-34* overexpression cells was significantly increased compared with control HeLa cells treated with ATO (Fig 5B, S20 and S21 Figs). These results suggest that *MLAA-34* overexpression increases the nuclear localization of β-catenin protein in HeLa cells treated with ATO.

## 4. Discussion

In our previous studies, we found that *MLAA-34* gene (GenBank no: AY288977.2) is a new anti-apoptotic gene related to acute monocytic leukemia and is a novel splice variant of *CAB39L*. At present, little is known about the function of *CAB39L* gene. Lo et al. found significantly higher expression of mRNA and protein of *CAB39L* in the tumor tissues of 37 patients with oral cancer compared with surrounding normal tissues [8]. A genome-wide association analysis and gene expression analysis found a considerable association between *CAB39L* and coffee addiction, and indicated that the protein encoded by *CAB39* may be involved in the metabolism of caffeine [9]. Rahmioglu et al. reported that *CAB39L* is a susceptibility gene for endometriosis and obesity [10]. These studies suggest that the function of *CAB39L* gene may be related to the metabolism of the substance, and it may be involved in the occurrence and development of tumors. Our previous study found that *MLAA-34* exhibits anti-apoptotic effects in U937 cells and clinical research has shown high expression of *MLAA-34* in primary and recurrent M5 patients and low expression in M5 patients with complete remission.

In our previous study, we generated U937 cells stably expressing *MLAA-34* and found that *MLAA-34* overexpression can significantly reduce the spontaneous apoptosis of U937 cells, increase the percentage of G_2_/M phase cells, and promote cell proliferation [4]. Co-immunoprecipitation, shotgun and bioinformatic analysis showed that the Wnt/β-catenin signaling pathway may be involved in the anti-apoptotic effect of *MLAA-34* in U937 cells. The present study found that when ATO induced apoptosis of U937 cells, the Wnt/β-catenin signaling pathway was inhibited and the expression of MLAA-34 protein was downregulated, suggesting that *MLAA-34* is involved in regulating the Wnt/β-catenin signaling pathway. To further confirm the anti-apoptotic effect of the *MLAA-34* gene, we investigated its function in another tumor cell line of non-hematologic tumors that does not express the *MLAA-34* gene.

Several studies have demonstrated that ATO significantly decreased HeLa cell survival and induced apoptosis and cell cycle arrest in G_2_/M phase [6, 11–15]. HeLa cells are very sensitive to ATO, with 1 μmol/L treatment producing apoptosis [6]. This dose has been very close to ATO in patients with acute myeloid leukemia in the treatment of dose [15], so as to induce HeLa cells apoptosis, the treatment concentration of ATO was set to 1μmol/L in our research. Although many studies have examined the mechanism of ATO inducing apoptosis in HeLa cells, the mechanism is still not clear and under discussion [16–21]. The inhibition of the expression of the HPV oncogenic gene *E6* by ATO may be one of the mechanisms [16]. In addition, *c-myc*, *Bc1-2* gene, a cell differentiation gene (*NDRG1*), intracellular ROS level, and the change of telomerase activity may be involved in the induction of apoptosis by ATO in HeLa cells [17–21]. Our results showed that inhibition of the Wnt/β-catenin signaling pathway may be one of the contributory mechanisms to ATO-induced apoptosis in HeLa cells.

Our study showed that overexpression of the *MLAA-34* gene had no effect on the growth, apoptosis and cell cycle of HeLa cells. Upon ATO treatment, the cell viability and colony formation ability of HeLa cells with *MLAA-34* overexpression were significantly higher than that of the control group, and the apoptosis rate and proportion of G_2_/M cells decreased. These results showed that the *MLAA-34* gene not only has anti-apoptotic effects in the acute monocytic leukemia cell line U937 but also in the HeLa cervical cancer cell line.

Our results showed that ATO treatment resulted in decreased expression of *β-catenin* mRNA and protein and the downstream target proteins c-Myc, cyclin B1, and cyclin D1 in HeLa cells. However, in *MLAA-34* stable cells, the ATO-mediated reduction of these factors was compromised, and the levels were significantly higher than that in the ATO-treated HeLa cells. Furthermore, we found that ATO-treated *MLAA-34* stable cells showed higher expression of nuclear *β-catenin* levels compared with HeLa cells treated with ATO. This suggested that the inhibitory effect of MLAA-34 protein on ATO-induced HeLa cell apoptosis may be partially achieved through activation of the Wnt/β-catenin signaling pathway.

Previous overexpression and RNA interference experiments have suggested that *MLAA-34* gene plays an antiapoptotic role in U937 cells with high expression of *MLAA-34*, and may play a role by participating in the Wnt/β-catenin signaling pathway. Recently, we have successfully expressed and purified MLAA-34 protein and isolated a fully human ScFv antibody (MA1) against MLAA-34 from a large human ScFv library. MA1 can not only specifically bind with U937 cells, but also inhibit the proliferation of U937 cells [22]. The results of this study showed that in the absence of *MLAA-34* expressing Hela cells, the expression of *MLAA-34* after exogenous vectors could also partially reduce ATO-induced apoptosis of HeLa cells, and the inhibitory effect of *MLAA-34* may be partially achieved through activation of the Wnt/β-catenin signaling pathway. In previous study, we found that the *MLAA-34* gene was highly expressed in U937 cells and expressed strongly in K562 and HL-60 cells, so we selected U937, K562 and HL-60 cells to do the knockdown experiments using RNA interference technique. The results showed that there was significant correlation between *MLAA-34* and the proliferation of K562 and HL-60 cells, the apoptosis rates of cells with siRNA infection were higher than that of control group. We found that MLAA-34-siRNA could induce the apoptosis of K562 and HL60 cells, it also significantly decreases the levels of β-catenin and TCF4, and so *MLAA-34* anti-apoptotic effect may be through the Wnt/β-catenin signaling pathway [5]. It is still not clear how *MLAA-34* activates and functions in the Wnt/β-catenin signaling pathway and whether the *MLAA-34* gene affects the Ras signaling pathway simultaneously.

## 5. Conclusions

Our results show that the Wnt/β-catenin signaling pathway is related to ATO-induced apoptosis in HeLa cells. The *MLAA-34* gene reduced ATO-induced apoptosis and G_2_/M arrest, and the anti-apoptotic effect may be achieved by activating the Wnt/β-catenin signaling pathway in HeLa cells.

## Supporting information

S1 Fig Expression of the *MLAA-34* mRNA in HeLa cells. The *MLAA-34* mRNA levels were determined in HeLa cell groups using RT-PCR. Lane 1. pGC-FU-MLAA-34 vector transfection group, Lane 2. PGC-FU vector transfection group, Lane 3. HeLa cells.
(TIF)

S2 Fig Expression of the *β-actin* mRNA in HeLa cells. The *β-actin* mRNA levels were determined in HeLa cell groups using RT-PCR. Lane 1. pGC-FU-MLAA-34 vector transfection group, Lane 2. PGC-FU vector transfection group, Lane 3. HeLa cells.
(TIF)

S3 Fig Expression of the MLAA-34 protein in HeLa cells. The MLAA-34 protein levels were determined in HeLa cell groups using western blot. Lane 1. pGC-FU-MLAA-34 vector transfection group, Lane 2. PGC-FU vector transfection group, Lane 3. HeLa cells.
(TIF)

S4 Fig Expression of the β-actin protein in HeLa cells. The β-actin protein levels were determined in HeLa cell groups using western blot. Lane 1. pGC-FU-MLAA-34 vector transfection group, Lane 2. PGC-FU vector transfection group, Lane 3. HeLa cells.
(TIF)

S5 Fig Expression of the nuclear β-catenin protein in U937 cells. Cells were treated with ATO (0, 1, 2, 4 μmol/L) for 48 h, and then harvested for analyses. The β-catenin protein levels were determined in nuclear extracts of U937 cells using western blot.
(TIF)

S6 Fig Expression of the nuclear H3 protein in U937 cells. Cells were treated with ATO (0, 1, 2, 4 μmol/L) for 48 h, and then harvested for analyses. The H3 protein levels were determined in nuclear extracts of U937 cells using western blot.
(TIF)

S7 Fig Expression of the MLAA-34 protein in U937 cells. Cells were treated with ATO (0, 1, 2, 4 μmol/L) for 48 h, and then harvested for analyses. The MLAA-34 protein levels were determined in U937 cells using western blot.
(TIF)

S8 Fig Expression of the c-Myc protein in U937 cells. Cells were treated with ATO (0, 1, 2, 4 μmol/L) for 48 h, and then harvested for analyses. The c-Myc protein levels were determined in U937 cells using western blot.
(TIF)

S9 Fig Expression of the cyclin B1 protein in U937 cells. Cells were treated with ATO (0, 1, 2, 4 μmol/L) for 48 h, and then harvested for analyses. The cyclin B1 protein levels were determined in U937 cells using western blot.
(TIF)

S10 Fig Expression of the cyclin D1 protein in U937 cells. Cells were treated with ATO (0, 1, 2, 4 μmol/L) for 48 h, and then harvested for analyses. The cyclin D1 protein levels were determined in U937 cells using western blot.
(TIF)

S11 Fig Expression of the β-actin protein in U937 cells. Cells were treated with ATO (0, 1, 2, 4 μmol/L) for 48 h, and then harvested for analyses. The β-actin protein levels were determined in U937 cells using western blot.
(TIF)

S12 Fig Effects of MLAA-34 on the levels of the *β-catenin* mRNA in HeLa cells. Cells were treated with ATO (1 μmol/L) for 48 h, and then harvested for analyses. RT-PCR analysis of *β-catenin* mRNA levels in all cell groups. 1: HeLa cells, 2: pGC-FU-MLAA-34 cells, 3: ATO+HeLa cells, 4: ATO+ pGC-FU-MLAA-34 cells.
(TIF)

S13 Fig Effects of MLAA-34 on the levels of the *β-actin* mRNA in HeLa cells. Cells were treated with ATO (1 μmol/L) for 48 h, and then harvested for analyses. RT-PCR analysis of *β-actin* mRNA levels in all cell groups. 1: HeLa cells, 2: pGC-FU-MLAA-34 cells, 3: ATO+HeLa cells, 4: ATO+ pGC-FU-MLAA-34 cells.
(TIF)

S14 Fig Effects of MLAA-34 on the levels of the β-catenin protein in HeLa cells. Cells were treated with ATO (1 μmol/L) for 48 h, and then harvested for analyses. Western blot of β-catenin protein levels in all cell groups. 1: HeLa cells, 2: pGC-FU-MLAA-34 cells, 3: ATO+HeLa cells, 4: ATO+ pGC-FU-MLAA-34 cells.
(TIF)

S15 Fig Effects of MLAA-34 on the levels of the β-actin protein in HeLa cells. Cells were treated with ATO (1 μmol/L) for 48 h, and then harvested for analyses. Western blot of β-actin protein levels in all cell groups. 1: HeLa cells, 2: pGC-FU-MLAA-34 cells, 3: ATO+HeLa cells, 4: ATO+ pGC-FU-MLAA-34 cells.
(TIF)

S16 Fig Effects of MLAA-34 on the levels of the c-Myc protein in HeLa cells. Cells were treated with ATO (1 μmol/L) for 48 h, and then harvested for analyses. Western blot of c-Myc protein levels in all cell groups. 1: HeLa cells, 2: pGC-FU-MLAA-34 cells, 3: ATO+HeLa cells, 4: ATO+ pGC-FU-MLAA-34 cells.
(TIF)

S17 Fig Effects of MLAA-34 on the levels of the cyclin B1 protein in HeLa cells. Cells were treated with ATO (1 μmol/L) for 48 h, and then harvested for analyses. Western blot of cyclin B1 protein levels in all cell groups. 1: HeLa cells, 2: pGC-FU-MLAA-34 cells, 3: ATO+HeLa cells, 4: ATO+ pGC-FU-MLAA-34 cells.
(TIF)

S18 Fig Effects of MLAA-34 on the levels of the cyclin D1 protein in HeLa cells. Cells were treated with ATO (1 μmol/L) for 48 h, and then harvested for analyses. Western blot of cyclin D1 protein levels in all cell groups. 1: HeLa cells, 2: pGC-FU-MLAA-34 cells, 3: ATO+HeLa cells, 4: ATO+ pGC-FU-MLAA-34 cells.
(TIF)

S19 Fig Effects of MLAA-34 on the levels of the β-actin protein in HeLa cells. Cells were treated with ATO (1 μmol/L) for 48 h, and then harvested for analyses. Western blot of β-actin protein levels in all cell groups. 1: HeLa cells, 2: pGC-FU-MLAA-34 cells, 3: ATO+HeLa cells, 4: ATO+ pGC-FU-MLAA-34 cells.
(TIF)

S20 Fig Effects of MLAA-34 on the levels of the nuclear β-catenin protein in HeLa cells. Cells were treated with ATO (1 μmol/L) for 48 h, and then harvested for analyses. The β-catenin protein levels were determined in nuclear extracts of Hela cells using western blot. 1: HeLa cells, 2: pGC-FU-MLAA-34 cells, 3: ATO+HeLa cells, 4: ATO+ pGC-FU-MLAA-34 cells.
(TIF)

S21 Fig Effects of MLAA-34 on the levels of the nuclear H3 protein in HeLa cells. Cells were treated with ATO (1 μmol/L) for 48 h, and then harvested for analyses. The H3 protein levels were determined in nuclear extracts of Hela cells using western blot. 1: HeLa cells, 2: pGC-FU-MLAA-34 cells, 3: ATO+HeLa cells, 4: ATO+ pGC-FU-MLAA-34 cells.
(TIF)
