# Supplementary figures and images for: Leukemia-associated gene MLAA-34 reduces arsenic trioxide-induced apoptosis in HeLa cells via activation of the Wnt/β-catenin signaling pathway
Source: PLoS One. 2017 Oct 23;12(10):e0186868. doi: 10.1371/journal.pone.0186868 (PMC5653344; doi:10.1371/journal.pone.0186868)

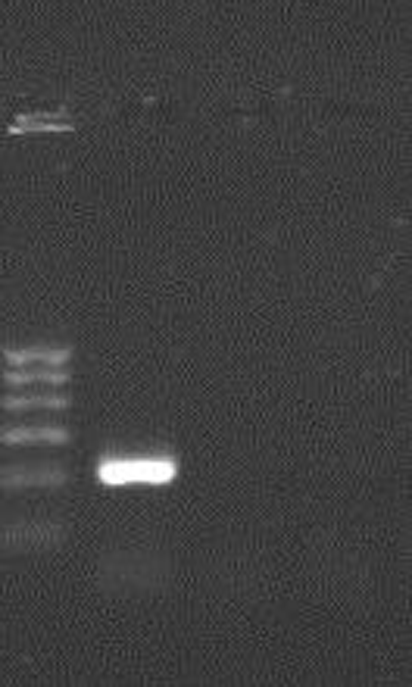

Supplement: S1 Fig — The MLAA-34 mRNA levels were determined in HeLa cell groups using RT-PCR. Lane 1. pGC-FU-MLAA-34 vector transfection group, Lane 2. PGC-FU vector transfection group, Lane 3. HeLa cells. (TIF) [file pone.0186868.s001.tif]

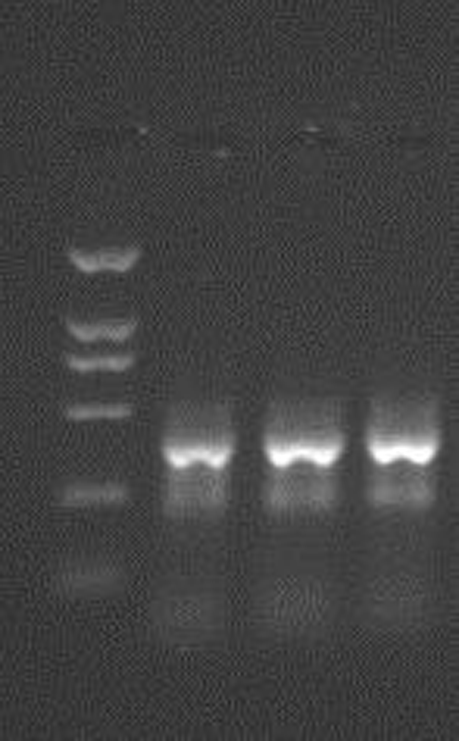

Supplement: S2 Fig — The β-actin mRNA levels were determined in HeLa cell groups using RT-PCR. Lane 1. pGC-FU-MLAA-34 vector transfection group, Lane 2. PGC-FU vector transfection group, Lane 3. HeLa cells. (TIF) [file pone.0186868.s002.tif]

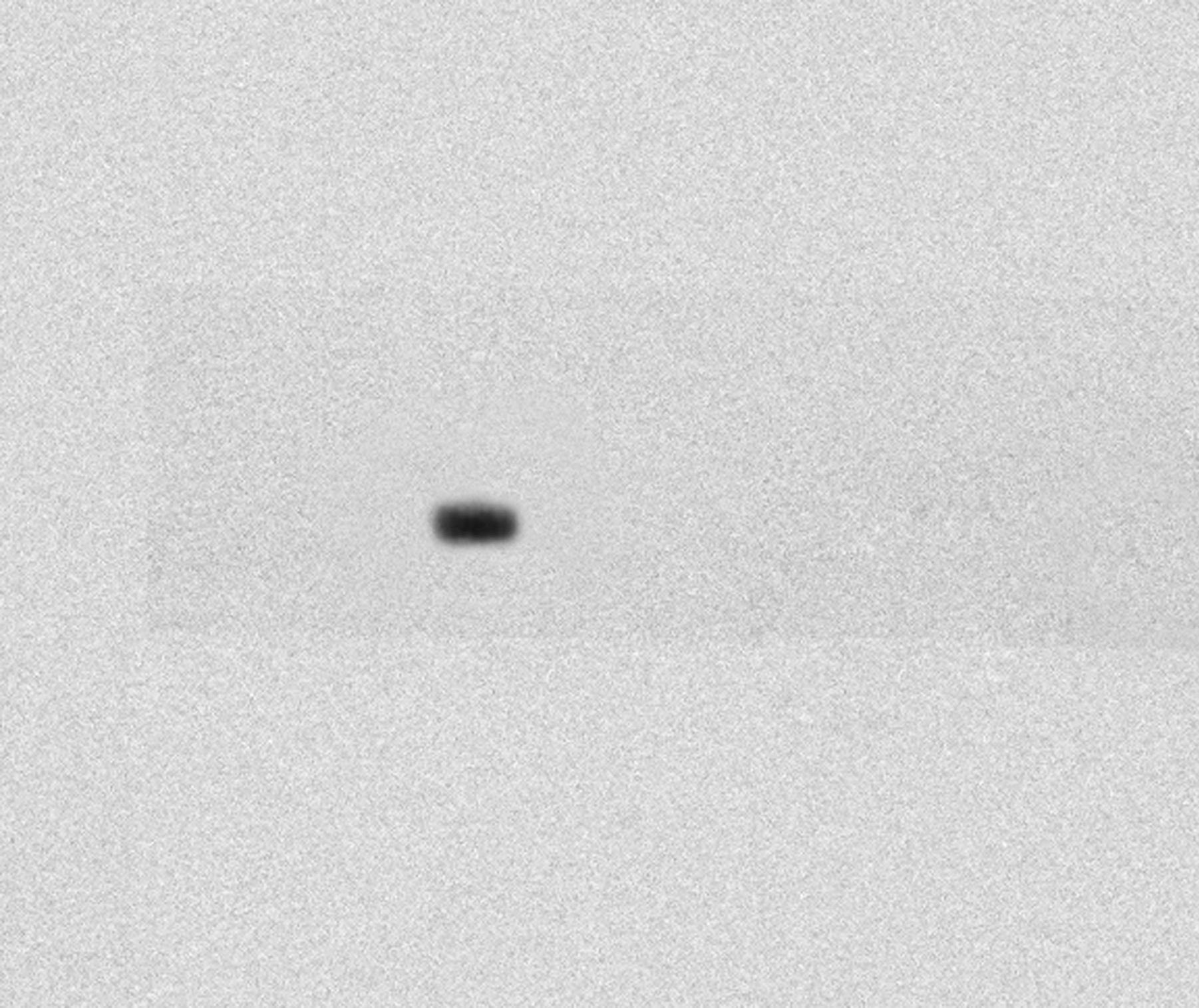

Supplement: S3 Fig — The MLAA-34 protein levels were determined in HeLa cell groups using western blot. Lane 1. pGC-FU-MLAA-34 vector transfection group, Lane 2. PGC-FU vector transfection group, Lane 3. HeLa cells. (TIF) [file pone.0186868.s003.tif]

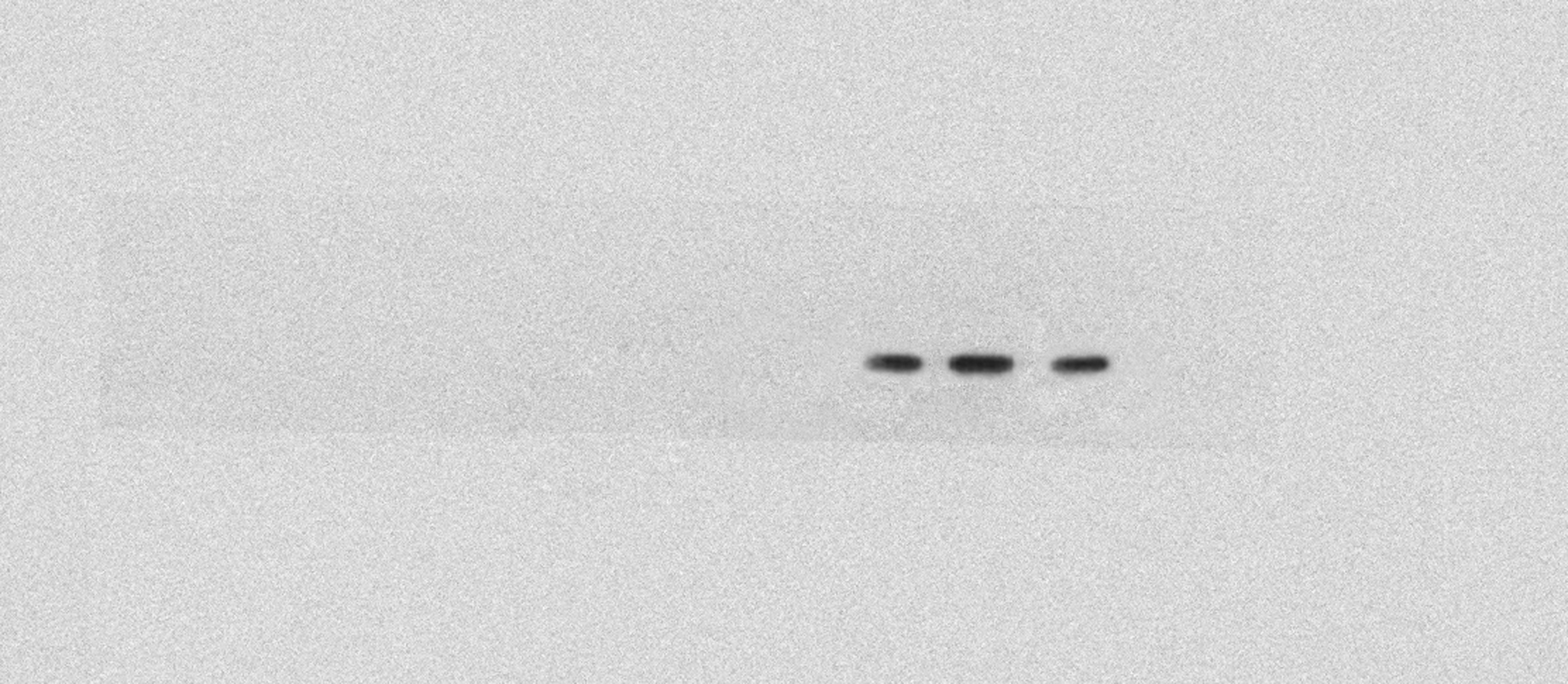

Supplement: S4 Fig — The β-actin protein levels were determined in HeLa cell groups using western blot. Lane 1. pGC-FU-MLAA-34 vector transfection group, Lane 2. PGC-FU vector transfection group, Lane 3. HeLa cells. (TIF) [file pone.0186868.s004.tif]

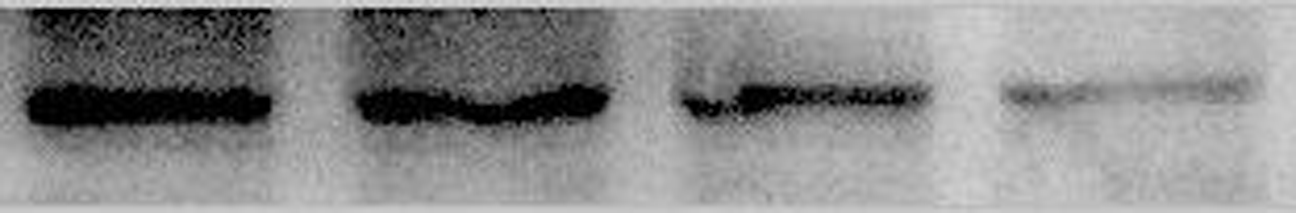

Supplement: S5 Fig — Cells were treated with ATO (0, 1, 2, 4 μmol/L) for 48 h, and then harvested for analyses. The β-catenin protein levels were determined in nuclear extracts of U937 cells using western blot. (TIF) [file pone.0186868.s005.tif]

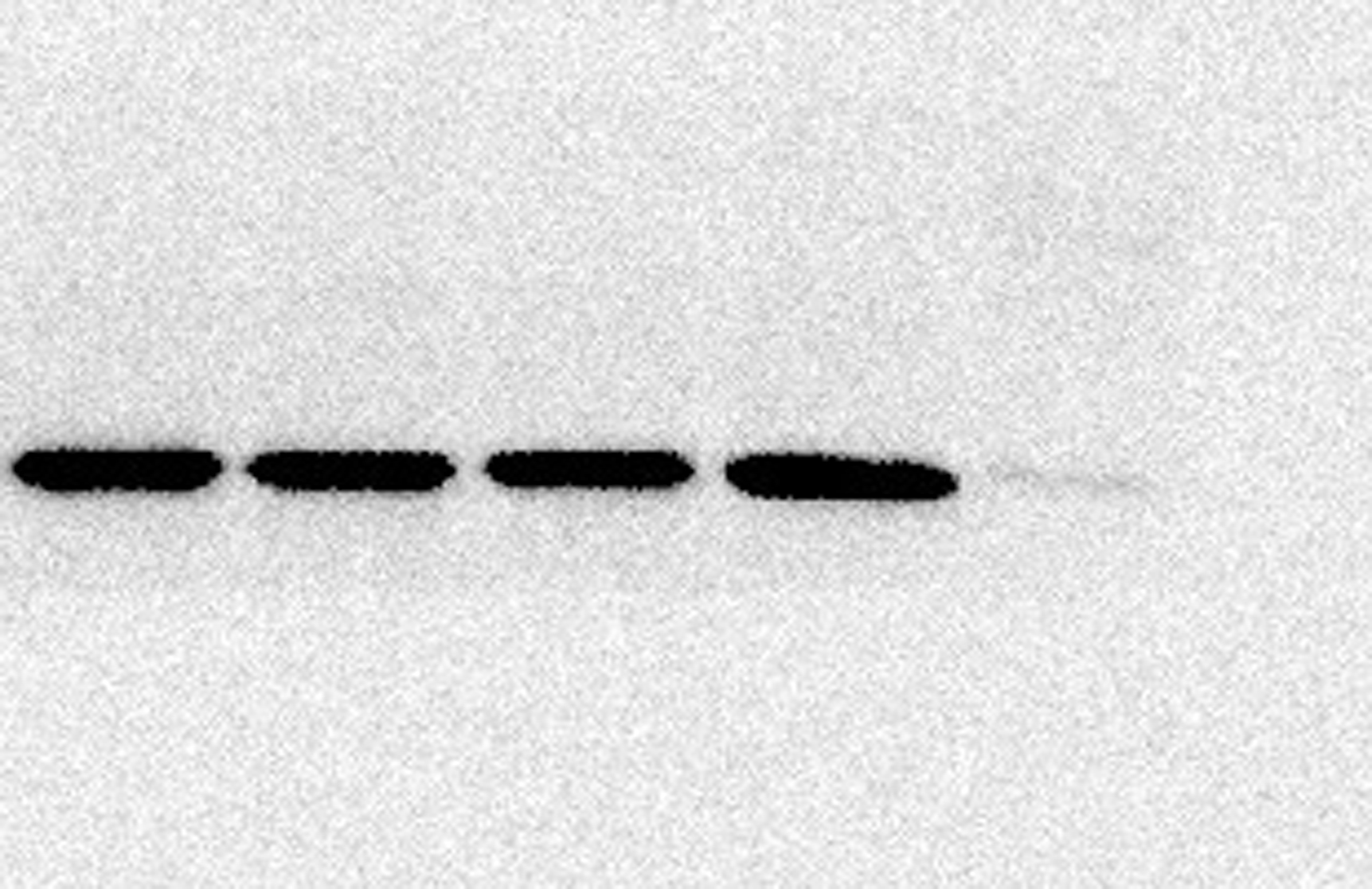

Supplement: S6 Fig — Cells were treated with ATO (0, 1, 2, 4 μmol/L) for 48 h, and then harvested for analyses. The H3 protein levels were determined in nuclear extracts of U937 cells using western blot. (TIF) [file pone.0186868.s006.tif]

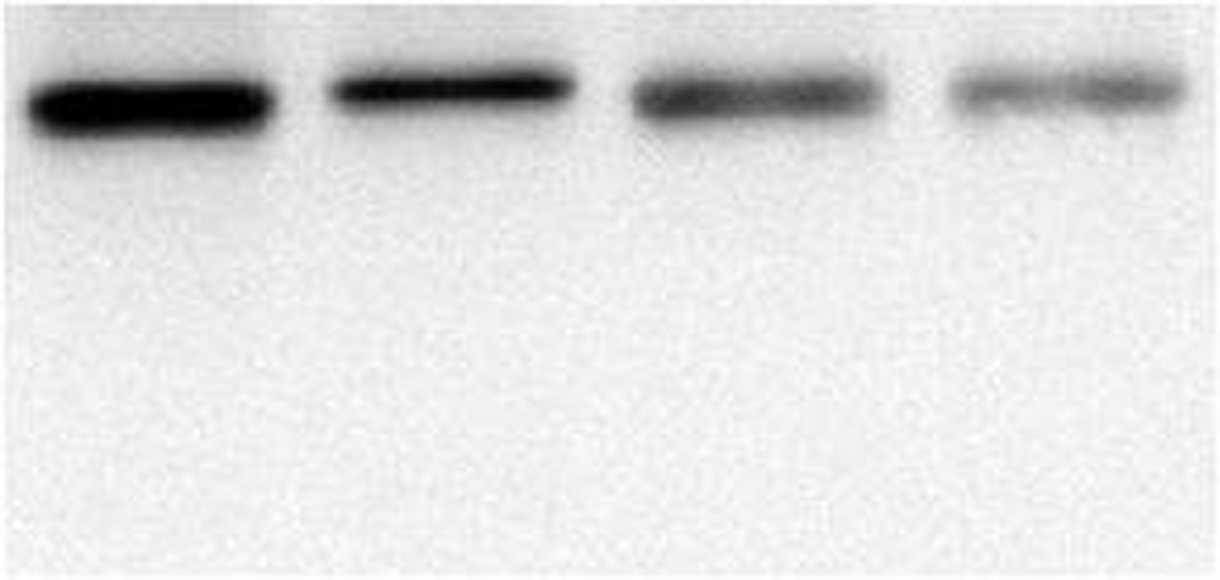

Supplement: S7 Fig — Cells were treated with ATO (0, 1, 2, 4 μmol/L) for 48 h, and then harvested for analyses. The MLAA-34 protein levels were determined in U937 cells using western blot. (TIF) [file pone.0186868.s007.tif]

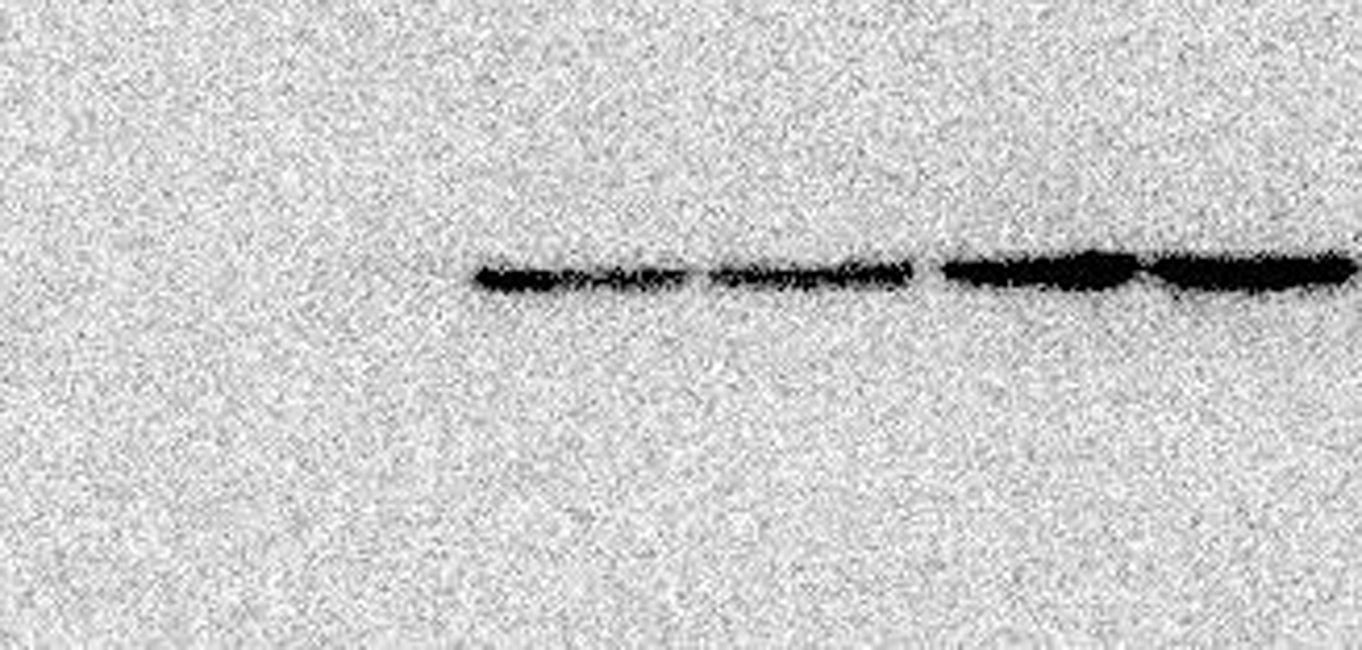

Supplement: S8 Fig — Cells were treated with ATO (0, 1, 2, 4 μmol/L) for 48 h, and then harvested for analyses. The c-Myc protein levels were determined in U937 cells using western blot. (TIF) [file pone.0186868.s008.tif]

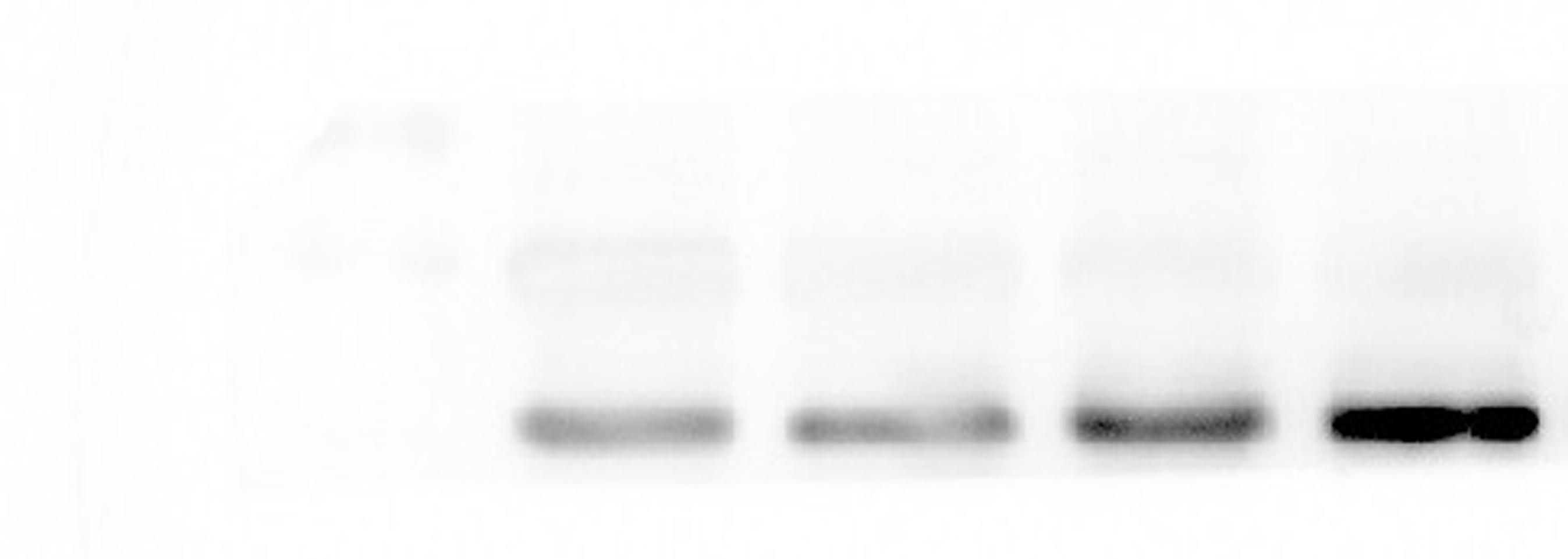

Supplement: S9 Fig — Cells were treated with ATO (0, 1, 2, 4 μmol/L) for 48 h, and then harvested for analyses. The cyclin B1 protein levels were determined in U937 cells using western blot. (TIF) [file pone.0186868.s009.tif]

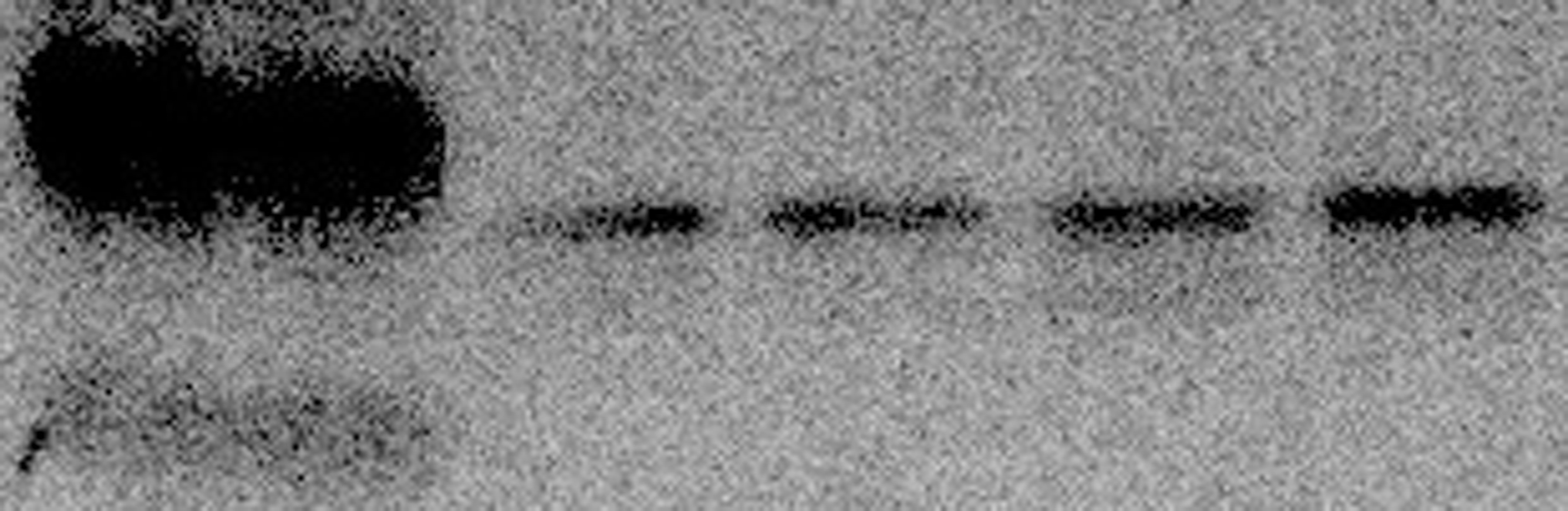

Supplement: S10 Fig — Cells were treated with ATO (0, 1, 2, 4 μmol/L) for 48 h, and then harvested for analyses. The cyclin D1 protein levels were determined in U937 cells using western blot. (TIF) [file pone.0186868.s010.tif]

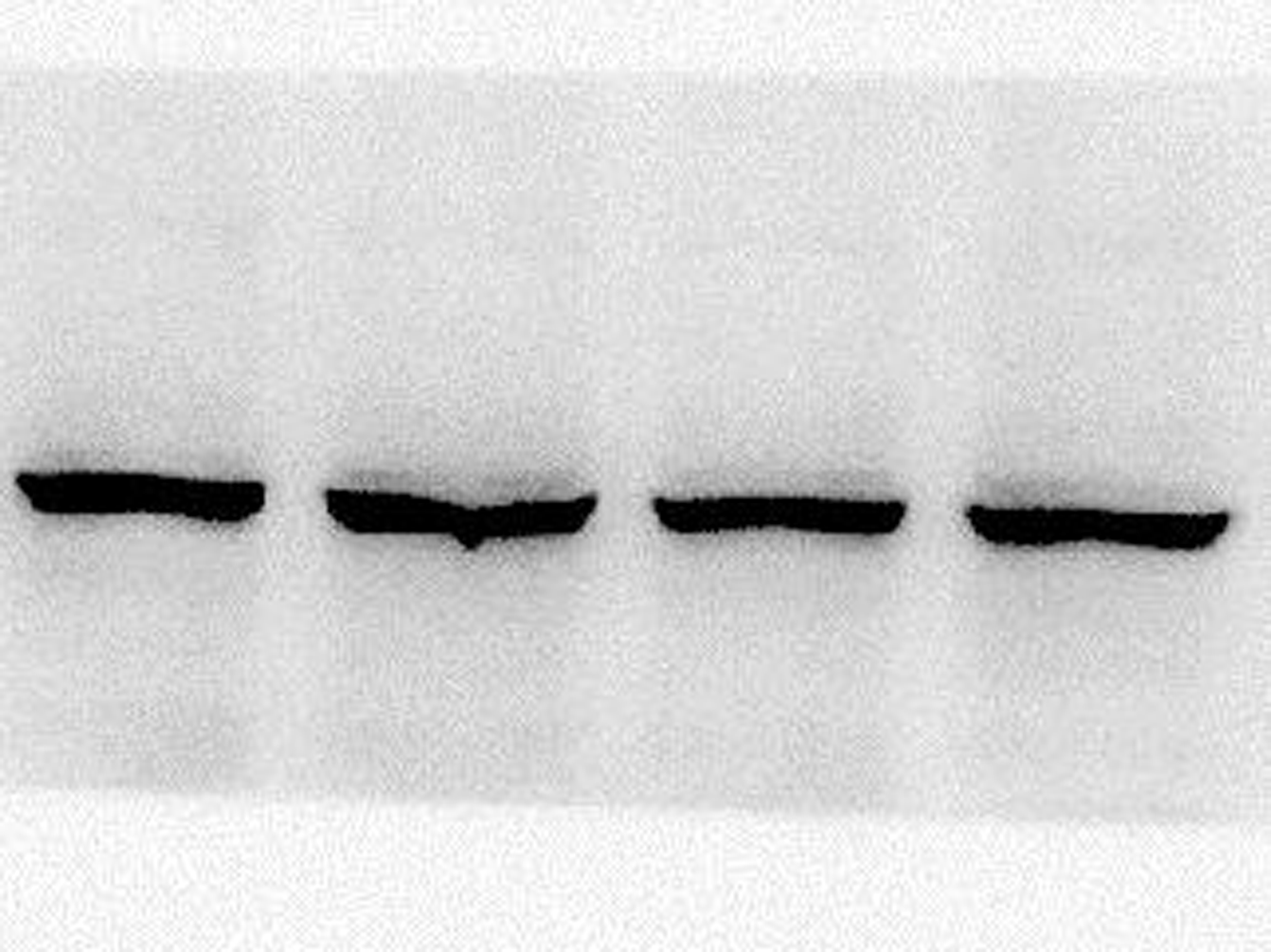

Supplement: S11 Fig — Cells were treated with ATO (0, 1, 2, 4 μmol/L) for 48 h, and then harvested for analyses. The β-actin protein levels were determined in U937 cells using western blot. (TIF) [file pone.0186868.s011.tif]

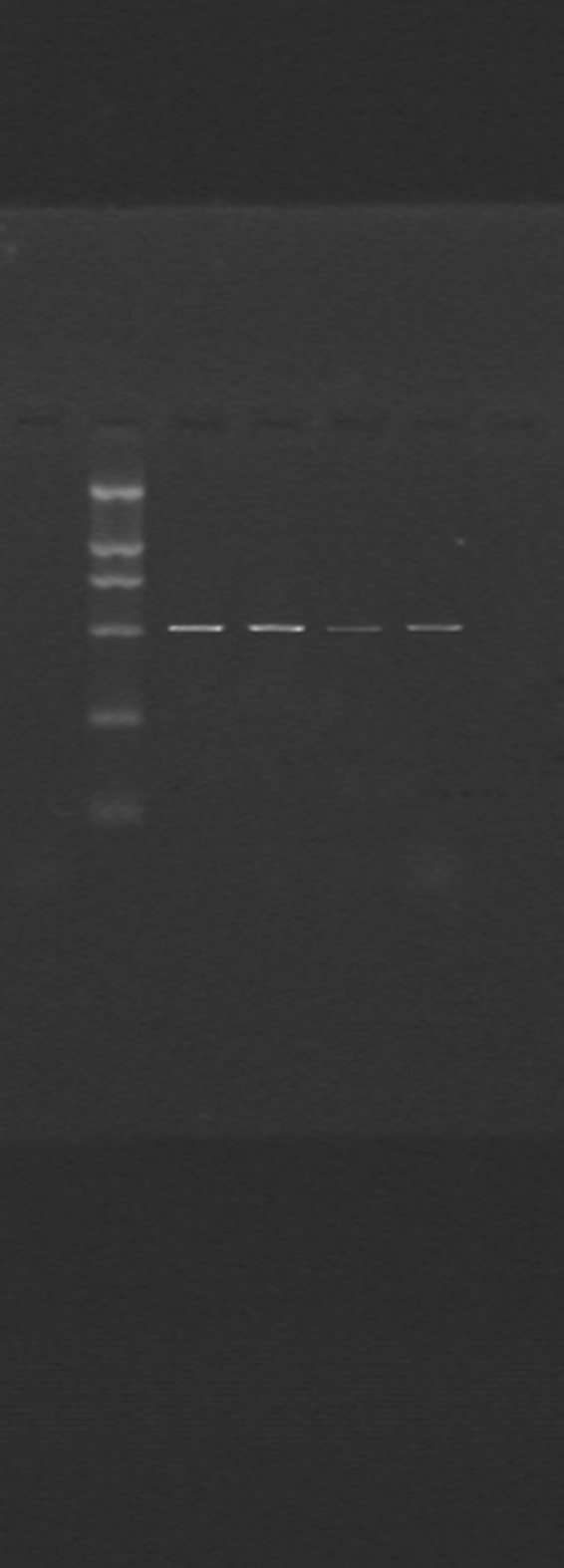

Supplement: S12 Fig — Cells were treated with ATO (1 μmol/L) for 48 h, and then harvested for analyses. RT-PCR analysis of β-catenin mRNA levels in all cell groups. 1: HeLa cells, 2: pGC-FU-MLAA-34 cells, 3: ATO+HeLa cells, 4: ATO+ pGC-FU-MLAA-34 cells. (TIF) [file pone.0186868.s012.tif]

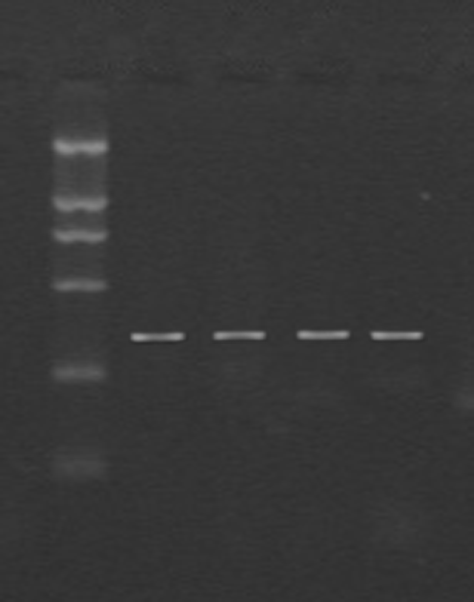

Supplement: S13 Fig — Cells were treated with ATO (1 μmol/L) for 48 h, and then harvested for analyses. RT-PCR analysis of β-actin mRNA levels in all cell groups. 1: HeLa cells, 2: pGC-FU-MLAA-34 cells, 3: ATO+HeLa cells, 4: ATO+ pGC-FU-MLAA-34 cells. (TIF) [file pone.0186868.s013.tif]

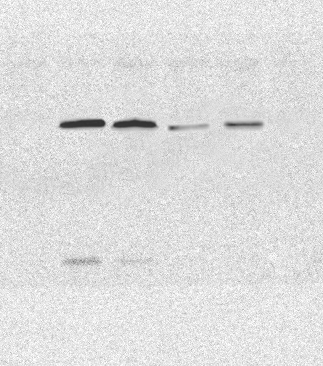

Supplement: S14 Fig — Cells were treated with ATO (1 μmol/L) for 48 h, and then harvested for analyses. Western blot of β-catenin protein levels in all cell groups. 1: HeLa cells, 2: pGC-FU-MLAA-34 cells, 3: ATO+HeLa cells, 4: ATO+ pGC-FU-MLAA-34 cells. (TIF) [file pone.0186868.s014.tif]

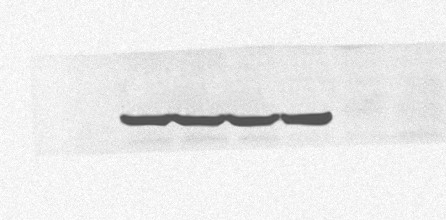

Supplement: S15 Fig — Cells were treated with ATO (1 μmol/L) for 48 h, and then harvested for analyses. Western blot of β-actin protein levels in all cell groups. 1: HeLa cells, 2: pGC-FU-MLAA-34 cells, 3: ATO+HeLa cells, 4: ATO+ pGC-FU-MLAA-34 cells. (TIF) [file pone.0186868.s015.tif]

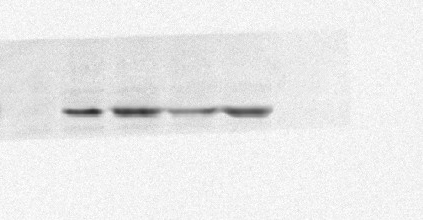

Supplement: S16 Fig — Cells were treated with ATO (1 μmol/L) for 48 h, and then harvested for analyses. Western blot of c-Myc protein levels in all cell groups. 1: HeLa cells, 2: pGC-FU-MLAA-34 cells, 3: ATO+HeLa cells, 4: ATO+ pGC-FU-MLAA-34 cells. (TIF) [file pone.0186868.s016.tif]

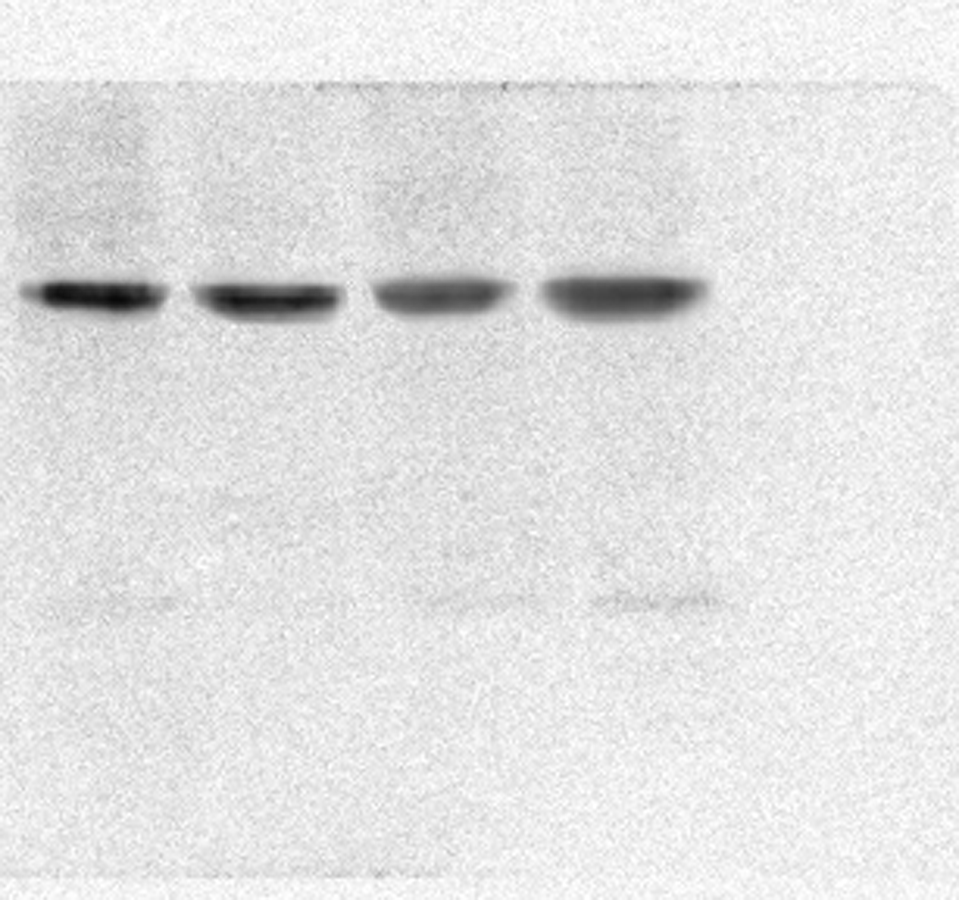

Supplement: S17 Fig — Cells were treated with ATO (1 μmol/L) for 48 h, and then harvested for analyses. Western blot of cyclin B1 protein levels in all cell groups. 1: HeLa cells, 2: pGC-FU-MLAA-34 cells, 3: ATO+HeLa cells, 4: ATO+ pGC-FU-MLAA-34 cells. (TIF) [file pone.0186868.s017.tif]

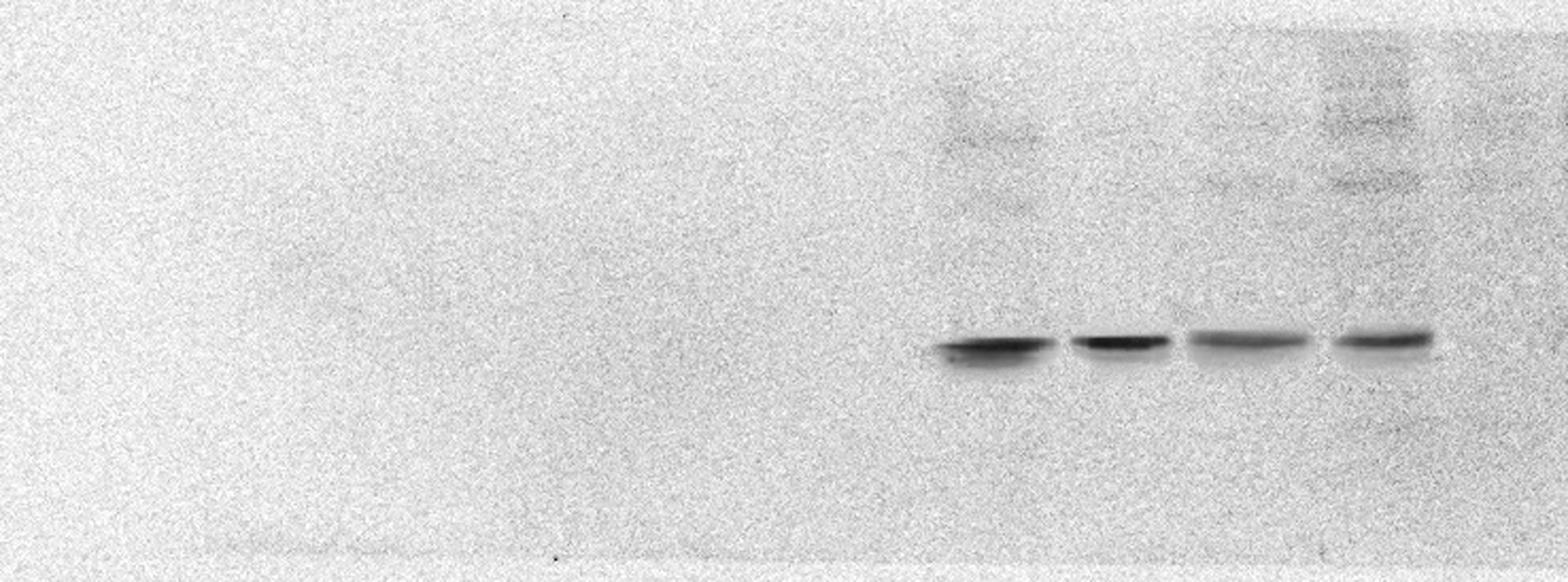

Supplement: S18 Fig — Cells were treated with ATO (1 μmol/L) for 48 h, and then harvested for analyses. Western blot of cyclin D1 protein levels in all cell groups. 1: HeLa cells, 2: pGC-FU-MLAA-34 cells, 3: ATO+HeLa cells, 4: ATO+ pGC-FU-MLAA-34 cells. (TIF) [file pone.0186868.s018.tif]

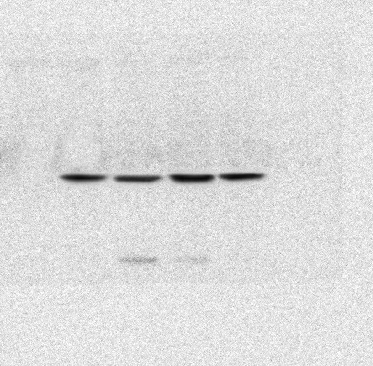

Supplement: S19 Fig — Cells were treated with ATO (1 μmol/L) for 48 h, and then harvested for analyses. Western blot of β-actin protein levels in all cell groups. 1: HeLa cells, 2: pGC-FU-MLAA-34 cells, 3: ATO+HeLa cells, 4: ATO+ pGC-FU-MLAA-34 cells. (TIF) [file pone.0186868.s019.tif]

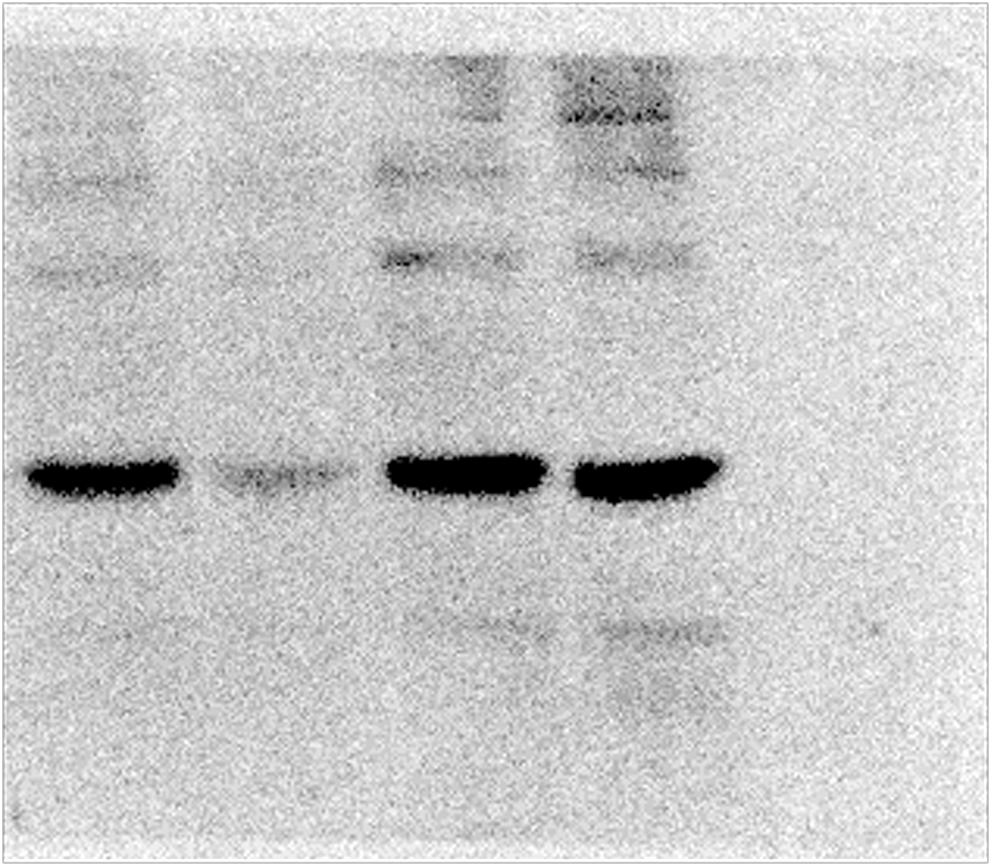

Supplement: S20 Fig — Cells were treated with ATO (1 μmol/L) for 48 h, and then harvested for analyses. The β-catenin protein levels were determined in nuclear extracts of Hela cells using western blot. 1: HeLa cells, 2: pGC-FU-MLAA-34 cells, 3: ATO+HeLa cells, 4: ATO+ pGC-FU-MLAA-34 cells. (TIF) [file pone.0186868.s020.tif]

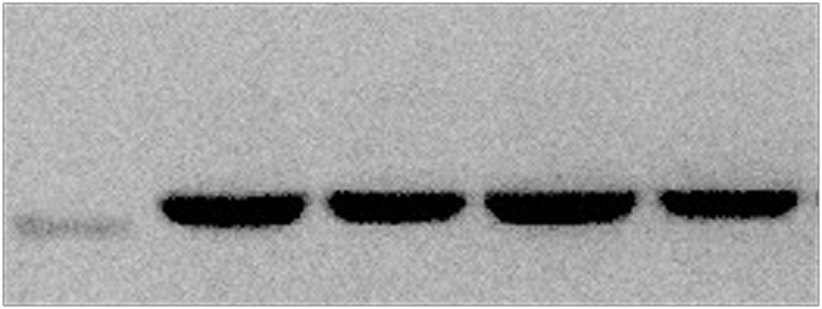

Supplement: S21 Fig — Cells were treated with ATO (1 μmol/L) for 48 h, and then harvested for analyses. The H3 protein levels were determined in nuclear extracts of Hela cells using western blot. 1: HeLa cells, 2: pGC-FU-MLAA-34 cells, 3: ATO+HeLa cells, 4: ATO+ pGC-FU-MLAA-34 cells. (TIF) [file pone.0186868.s021.tif]
